# 4-Hydroxynonenal induces apoptosis in human osteoarthritic chondrocytes: the protective role of glutathione-S-transferase

**DOI:** 10.1186/ar2503

**Published:** 2008-09-09

**Authors:** France Vaillancourt, Hassan Fahmi, Qin Shi, Patrick Lavigne, Pierre Ranger, Julio C Fernandes, Mohamed Benderdour

**Affiliations:** 1 Orthopaedic Research Laboratory, Hôpital du Sacré-Cæur de Montréal, Department of Surgery, University of Montreal, 5400 Gouin Blvd. West, Montreal, QC, H4J 1C5, Canada

## Abstract

**Introduction:**

4-Hydroxynonenal (HNE) is one of the most abundant and reactive aldehydes of lipid peroxidation products and exerts various effects on intracellular and extracellular signalling cascades. We have previously shown that HNE at low concentrations could be considered as an important mediator of catabolic and inflammatory processes in osteoarthritis (OA). In the present study, we focused on characterizing the signalling cascade induced by high HNE concentration involved in cell death in human OA chondrocytes.

**Methods:**

Markers of apoptosis were quantified with commercial kits. Protein levels were evaluated by Western blotting. Glutathione (GSH) and ATP levels were measured with commercial kits. Glucose uptake was assessed by 2-deoxy-D-[^3^H]-glucose. The role of GSH-S-transferase A4-4 (GSTA4-4) in controlling HNE-induced chondrocyte apoptosis was investigated by chondrocyte transfection with small interfering RNA (siRNA) or with the expression vector of GSTA4-4.

**Results:**

Our data showed that HNE at concentrations of up to 10 μM did not alter cell viability but was cytotoxic at concentrations of greater than or equal to 20 μM. HNE-induced chondrocyte death exhibited several classical hallmarks of apoptosis, including caspase activation, cytochrome *c *and apoptosis-induced factor release from mitochondria, poly (ADP-ribose) polymerase cleavage, Bcl-2 downregulation, Bax upregulation, and DNA fragmentation. Our study of signalling pathways revealed that HNE suppressed pro-survival Akt kinase activity but, in contrast, induced Fas/CD95 and p53 expression in chondrocytes. All of these effects were inhibited by an antioxidant, *N*-acetyl-cysteine. Analysis of cellular energy and redox status showed that HNE induced ATP, NADPH, and GSH depletion and inhibited glucose uptake and citric acid cycle activity. GSTA4-4 ablation by the siRNA method augmented HNE cytotoxicity, but, conversely, its overexpression efficiently protected chondrocytes from HNE-induced cell death.

**Conclusion:**

Our study provides novel insights into the potential mechanisms of cell death in OA cartilage and suggests the potential role of HNE in OA pathophysiology. GSTA4-4 expression is critically important for cellular defence against oxidative stress-induced cell death in OA cartilage, possibly by HNE elimination.

## Introduction

Osteoarthritis (OA) is a degenerative disease characterized by the loss and abnormal remodelling of cartilage extracellular matrix (ECM) [1,2]. Changes in matrix quality stem from the failure of chondrocytes to maintain a balance between protein synthesis and degradation. Chondrocytes are the only cell type found in mature cartilage, and their death may contribute to metabolic and structural changes in OA cartilage. Depending upon the region being examined, cartilage may be devoid of chondrocytes, presumably as a result of cell death, or contain clusters of chondrocytes that have undergone division, possibly in response to ECM depletion [2]. The superficial zones of OA cartilage contain empty lacunae, fragmented chondrocytes, and nuclear condensation [3]. Several studies have revealed increased apoptotic cell death in lesional areas compared with non-lesional areas in cartilage from the same OA patient, while apoptotic cells are rarely seen in normal cartilage [4]. In an experimental mouse model of OA, it has been shown that chondrocyte apoptosis in articular cartilage is correlated with age and disease severity [5]. In addition to cells with ultrastructural features of apoptosis, human OA cartilage contains cells that appear necrotic. Since cartilage does not have mononuclear phagocytes and is avascular, dead cells or apoptotic bodies are not removed but remain in the lacunae, where they disintegrate and release their contents. Ultrastructural evidence suggests that disintegration of chondrocytes in articular cartilage may lead to the formation of membrane-enclosed structures resembling matrix vesicles [3,6]. These structures, which are remnants of dead cells, may in fact be apoptotic bodies and may contribute to matrix mineralization or degradation in OA.

Oxidative stress has been at the centre of the pathophysiological scene of OA disease over the past 30 years. Alterations in mitochondrial redox metabolism and respiratory functions may elicit the increased production of reactive oxygen species (ROS) in chondrocytes. Besides causing degradation [7] or inhibiting the synthesis of cartilage matrix [8], ROS may induce chondrocyte apoptosis. Nitric oxide (NO) has long been considered as the primary inducer of chondrocyte apoptosis mediated by caspase-3 and tyrosine kinase activation [9]. However, it has become clear that NO by itself cannot initiate apoptosis and that the concomitant production of superoxide anion is required [10], indicating a role for peroxinitrite in this process. In addition, there is a significant correlation between the level of NO generation and the prevalence of apoptotic cells in cartilage tissue during experimentally induced OA in rabbits [11].

Aldehydes are produced from ROS- and NO-induced lipid peroxidation (LPO) of membrane polyunsaturated fatty acids. Similar to free radicals, aldehydes are electrophiles that bind to nucleophilic groups of proteins, (amino)phospholipids, and nucleic acids, but their relatively longer half-life makes them candidates for the propagation of damage to neighbouring cells. Among the aldehydes, 4-hydroxy-2-alkenals, such as 4-hydroxynonenal (HNE), are considered to be the most reactive species because of their α, β-double bond [12]. This aldehyde is highly reactive with a variety of biomolecules, such as proteins, lipids, and nucleic acids, contributing to the pathogenesis of human chronic diseases [13]. Like ROS, HNE can also induce, in many cell types of different origins, various biological effects, such as alterations in cell proliferation, cell cycle procession, and apoptosis [14,15]. Studies have shown that antioxidant agents such as *N*-acetyl-cysteine (NAC) or glutathione-S-transferase A4-4 (GSTA4-4) overexpression suppress HNE production and inhibit the apoptotic process in several cell lines induced by this aldehyde [16,17]. In a recent study, we observed that the level of HNE protein adducts is higher in OA synovial fluid compared with normal subjects [18]. Moreover, we have demonstrated that, in OA cartilage, HNE can induce transcriptional as well as post-translational modifications of type II collagen (Col II) and matrix metalloproteinase-13 (MMP-13), resulting in cartilage ECM degradation. Additionally, HNE can selectively induce cyclooxygenase-2 (COX-2) expression via ATF/CRE (activating transcription factor/cAMP response element) activation and inhibit the inducible form of NO synthase (iNOS) via nuclear factor-kappa B (NF-κB) inactivation in human chondrocytes [19]. The objective of this study, which expands on our previous works, was to investigate the mechanism of HNE-induced apoptotic cell death in human OA chondrocytes. In addition, we evaluated the role of NAC treatment and GSTA4-4 overexpression in the protection against chondrocyte apoptosis induced by HNE.

## Materials and methods

### Specimen selection and chondrocyte culture

Cartilage specimens were obtained from OA patients who underwent total knee arthroplasty (64 ± 9 years, mean ± standard error, n = 34). Diagnoses were established according to American College of Rheumatology criteria [20]. OA cartilage (femoral condyles and tibial plateaus) was obtained under aseptic conditions and carefully dissected from the underlying bone in each specimen. This project and the informed consent form were approved by the institutional Ethics Committee Board of the Hôpital du Sacré-Coeur de Montréal. OA chondrocytes were released from cartilage explants as described previously [18]. Isolated chondrocytes were seeded at high density in culture flasks until confluence in Dulbecco's modified Eagle's medium (DMEM) supplemented with 10% heat-inactivated fetal bovine serum (FBS) and 100 U/mL penicillin/100 mg/mL streptomycin (Invitrogen Canada Inc, Burlington, ON, Canada). First-passage chondrocytes were seeded in culture plates at 10^5 ^cells/cm^2 ^and incubated for 48 hours in the above medium. Before the experiments, the medium was replaced by fresh medium containing 2% FBS and treated as indicated in the experimental protocols.

### Cell viability

HNE-induced chondrocyte cytotoxicity was evaluated by MTT (3-[4,5-dimethylthiazol-2-yl]-2,5-diphenyltetrazolium bromide) assay [21]. Tests were performed in 96-well plates. Briefly, chondrocytes were incubated for 16 hours with increasing concentrations (0 to 30 μM) of HNE (Cayman Chemical Company, Ann Arbor, MI, USA) or with 30 μM HNE for increasing times of incubation in the presence or absence of 200 μM NAC (Sigma-Aldrich, Oakville, ON, Canada). To explore the signalling cascade in HNE-induced cell death, cells were incubated for 1 hour with the inhibitor of poly (ADP-ribose) polymerase-1 (PARP-1), 5-iodo-6-amino-1,2-benzopyrone, at 50 and 100 μM (INH_2_BP; Sigma-Aldrich) or with anti-Fas/CD95 antibody at 20 μg/mL, followed by another incubation for 16 hours with 30 μM HNE. Then, the cells were incubated with 0.5 mg/mL MTT for 15 minutes at 37°C. Thereafter, 100 μL of solubilization solution (0.04 M HCl-isopropanol) was added. The amount of MTT formazan product was quantified by measuring of optical density at 570 nm with a microplate reader (BioTek Instruments, Winooski, VT, USA).

### Nuclear morphology study for apoptosis

Apoptotic nuclear morphology was assessed by Hoechst 33258 incorporation (Sigma-Aldrich). Briefly, chondrocytes (10^5 ^cells/cm^2^) were treated with or without 30 μM HNE for 16 hours. The cells were fixed with 4% paraformaldehyde at room temperature for 15 minutes and then washed and stained with 10 μg/mL Hoechst 33258 in phosphate-buffered saline (PBS) at room temperature for 10 minutes. Hoechst-stained cells were analyzed by fluorescence microscopy.

### Measurement of caspase activities

Enzymatic caspase-8, -9, and -3 activities were measured with commercial kits. Chondrocytes (10^5 ^cells/cm^2^) were treated with 30 μM HNE for increasing incubation times (0 to 16 hours). To measure caspase-8 and -9 activities, the cells were washed with PBS and resuspended in 100 μL of lysis buffer (R&D Systems, Minneapolis, MN, USA), left on ice for 10 minutes, and centrifuged. Protein concentration of the supernatants was measured according to the bicinchoninic acid method (Pierce, Rockford, IL, USA). Total proteins (50 μg) were reacted with 200 μM IETD-*p*NA or LEHD-*p*NA substrate in the presence of 100 μL of reaction buffer. To quantitate caspase-3 activity, the cells were washed with PBS and lysed in 100 μL of lysis buffer (Sigma-Aldrich), left on ice for 15 minutes, and centrifuged. Total proteins (5 μg) were reacted with 200 μM DEVD-*p*NA substrate in the presence of 100 μL of reaction buffer. After 16 hours of incubation at 37°C, *p*-nitroanilide release was measured at 405 nm for caspase-3, -8, and -9.

### Quantitation of Bcl-2

Chondrocytes (10^5 ^cells/cm^2^) were treated with 30 μM HNE for increasing incubation times (0 to 16 hours). The protein expression of the antiapoptotic Bcl-2 was assayed in cell extracts with a Bcl-2 enzyme-linked immunosorbent assay (ELISA) kit (catalogue number QIA23; Calbiochem, now part of EMD Biosciences, Inc., San Diego, CA, USA) according to the manufacturer's instructions. The Bcl-2 level was expressed in units per milligram of protein.

### Measurement of DNA fragmentation

Cytoplasmic histone-associated DNA fragments were quantitated with a Cell Death Detection ELISA^PLUS ^kit (Roche Applied Science, Laval, QC, Canada) according to the manufacturer's recommendations. Briefly, chondrocytes (2 × 10^6 ^cells) were treated for 16 hours with increasing HNE concentrations (0 to 30 μM) with or without 200 μM NAC. After incubation, the cells were lysed with lysis buffer for 30 minutes and centrifuged at 200 *g *for 10 minutes. The supernatant and a mixture of anti-histone-biotin and anti-DNA-peroxidase were added to streptavidin-coated microplates and incubated for 2 hours at room temperature. After adding the substrate, absorbance was measured at 405 nm.

### Quantitation of cytochrome c release in cytosolic fractions

To measure cytochrome *c *release, chondrocytes (1 × 10^6 ^cells) were treated for 16 hours with increasing HNE concentrations (0 to 30 μM) with or without 200 μM NAC, washed with PBS, and resuspended in 1 mL of buffer (220 mM mannitol, 70 mM sucrose, 10 mM Hepes KOH, pH 7.4, 10 mM ethylenediaminetetraacetic acid [EDTA], 10% glycerol). The cell suspension was incubated on ice for 15 minutes and centrifuged at 10,000 *g *for 15 minutes. The pellet containing the mitochondria was resuspended in 200 μL of the above buffer and served to assess citric acid activity as described below. Cytochrome *c *level was measured in cytosolic fractions (supernatants) with a Cytochrome *c *ELISA kit (EMD Biosciences, Inc.) according to the manufacturer's directions. Cytochrome *c *level was expressed in nanograms per milligram of protein.

### Citric acid cycle activity

Mitochondrial NADP^+^-dependent isocitrate dehydrogenase (mNADP^+^-ICDH) activity was assessed in mitochondrial fractions prepared, as described above, from chondrocytes treated with 30 μM HNE for 16 hours. The assay was performed in the presence of 5 mM isocitrate, 1 mM NADP, and 2 mM MgCl_2_. Activities were expressed in units per milligram of protein, where 1 unit was defined as the amount of enzyme catalyzing the conversion of 1 μmol substrate per minute at 37°C.

### Quantitation of ATP level

ATP level was assessed in cellular extracts from chondrocytes treated with 30 μM HNE for 16 hours with an ATP Assay kit from EMD Biosciences, Inc. The results were expressed as picomoles per milligram of proteins.

### Quantification of reduced glutathione and oxidized glutathione levels

Chondrocytes (2 × 10^6 ^cells) were incubated for increasing time periods (0 to 16 hours) with 30 μM HNE. The cells were washed with PBS and centrifuged at 800 *g *for 5 minutes. Pellets were resuspended in buffer (0.4 M 2-[*N*-morpholino] ethanesulphonic acid, 0.1 M phosphate, 2 mM EDTA) and centrifuged at 10,000 *g *for 15 minutes. Glutathione (GSH) and oxidized GSH (GSSG) levels were quantified with a Glutathione Assay Kit (Cayman Chemical Company) according to the manufacturer's directions. Values were expressed as the GSSG/[GSSG+GSH] ratio.

### Western blot analysis

Chondrocytes were incubated for increasing time periods (0 to 16 hours) with 30 μM HNE or with increasing concentrations of HNE (0 to 30 μM) for 16 hours with or without 200 μM NAC. Cellular protein extract (20 μg) or nuclear protein (5 μg), isolated as previously described [19], was subjected to discontinuous 4% to 12% SDS-PAGE as previously described [18]. The primary antibodies used were rabbit anti-human PARP, rabbit anti-human apoptosis-inducing factor (AIF), rabbit anti-human Bax, rabbit anti-human caspase-3, -9, anti-Akt (EMD Biosciences, Inc.), rabbit anti-human caspase-8, anti-p53, and mouse anti-human Fas/CD95 (Santa Cruz Biotechnology, Santa Cruz, CA, USA), and anti-GSTA4-4 (Abnova, Taipei, Taiwan). After serial washes, primary antibodies were detected by goat anti-rabbit IgG or goat anti-mouse IgG conjugated to horseradish peroxidase (Jackson ImmunoResearch Laboratories, Inc., West Grove, PA, USA). Specific signals were visualized with enhanced chemiluminescence detection kits (Pierce).

### Plasmids and transient GSTA4-4 transfection

GSTA4-4 small interfering RNA (siRNA) and randomly sequenced siRNA as negative controls were purchased from Ambion (Austin, TX, USA). Wild-type and mutant GSTA4-4 expression plasmids were generously provided by Dr Sanjay Awasthi [17] (University of North Texas Health Science Center, Fort Worth, TX, USA). Subconfluent chondrocytes were transiently transfected by Lipofectamine 2000™ reagent (Invitrogen Canada Inc) according to the manufacturer's protocol. Briefly, transfections were conducted for 6 hours with DNA lipofectamine complexes containing 10 μL of lipofectamine reagent, 100 nM GSTA4-4 siRNA (a mixture of three siRNAs targeting the GSTA4-4 gene) or randomly sequenced siRNA, or 2 μg of DNA plasmid and 0.5 μg of pCMV-β-gal (as a control of transfection efficiency). After washing, experiments were performed in 2% FBS fresh medium supplemented or not supplemented with 30 μM HNE. Then, cell viability and GSTA4-4 expression were analyzed by the MTT method and Western blotting, respectively, as described previously. β-gal level was measured with ELISA kits from Roche Diagnostics Canada (Laval, QC, Canada).

### Glucose uptake

Chondrocytes were cultured for 16 hours in 24-well plates at 5 × 10^5 ^cells per well in 2% FBS/DMEM in the presence or absence of 30 μM HNE. The culture media were replaced by 2% FBS/glucose-free DMEM containing 10 μCi/mL 2-deoxy-D-[^3^H]-glucose. Then, plates were incubated for 20 minutes at 37°C. Subsequently, the media were aspirated and the cells were washed three times with cold PBS. They were then lysed with 400 μL/well of cell death lysis buffer (EMD Biosciences, Inc.) for 15 minutes. Volumes of 300 μL of cell lysates were transferred to scintillation vials, and radioactivity was measured by scintillation counting. The data are expressed as counts per minute (cpm) per milligram of proteins.

### Statistical analysis

Results were expressed as the mean ± standard error of the mean of eight specimens, and assays were performed in three independent experiments. Statistical analysis was performed using the two-tailed paired Student *t *test, and a difference of less than or equal to 0.05 was considered significant.

## Results

### HNE caused cell death in human osteoarthritis chondrocytes

**Figure 1:**
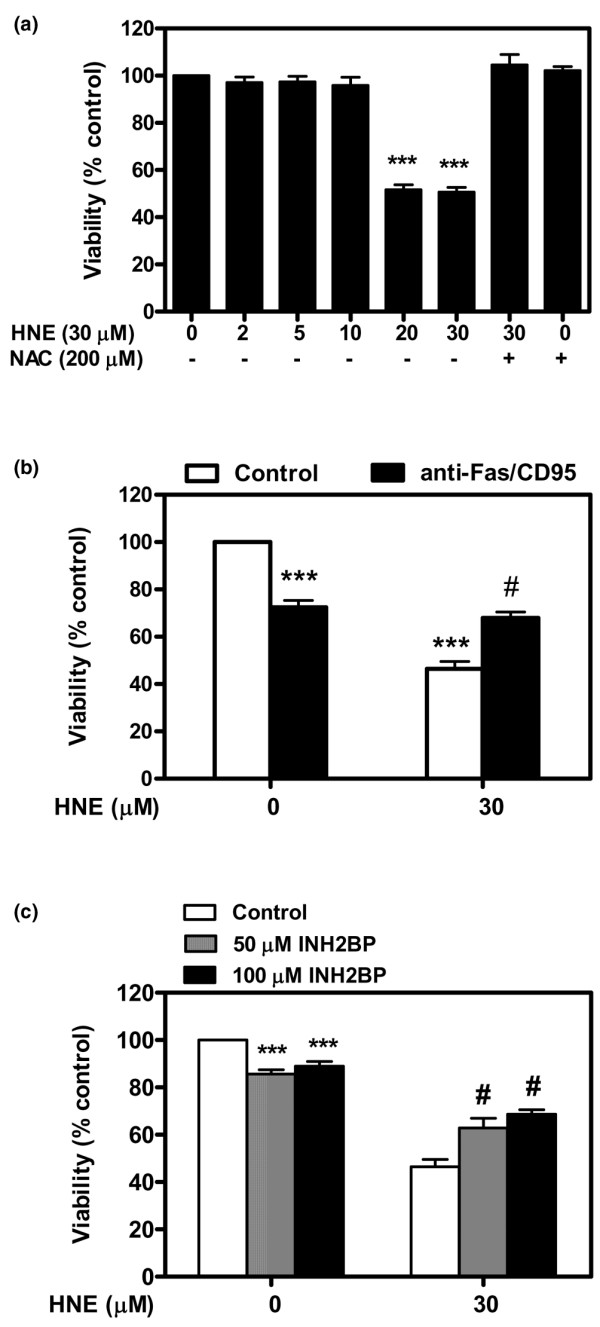
4-Hydroxynonenal (HNE)-induced cell death. **(a) **Chondrocytes were pre-incubated for 1 hour with or without 200 μM *N*-acetyl-cysteine (NAC) followed by another incubation for 16 hours with increasing concentrations of HNE (0 to 30 μM). Chondrocytes were pre-incubated for 1 hour with or without **(b) **20 μg/mL anti-Fas/CD95 antibody or **(c) **50 and 100 μM INH_2_BP followed by another incubation for 16 hours with or without 30 μM HNE. Cell viability was evaluated by MTT assay. Data are mean ± standard error of the mean (n = 8). Statistics: Student unpaired *t *test; ***P < 0.001 (30 μM HNE versus untreated cells), ^#^*P *< 0.05 (30 μM HNE+inhibitor versus 30 μM HNE).

**Figure 2:**
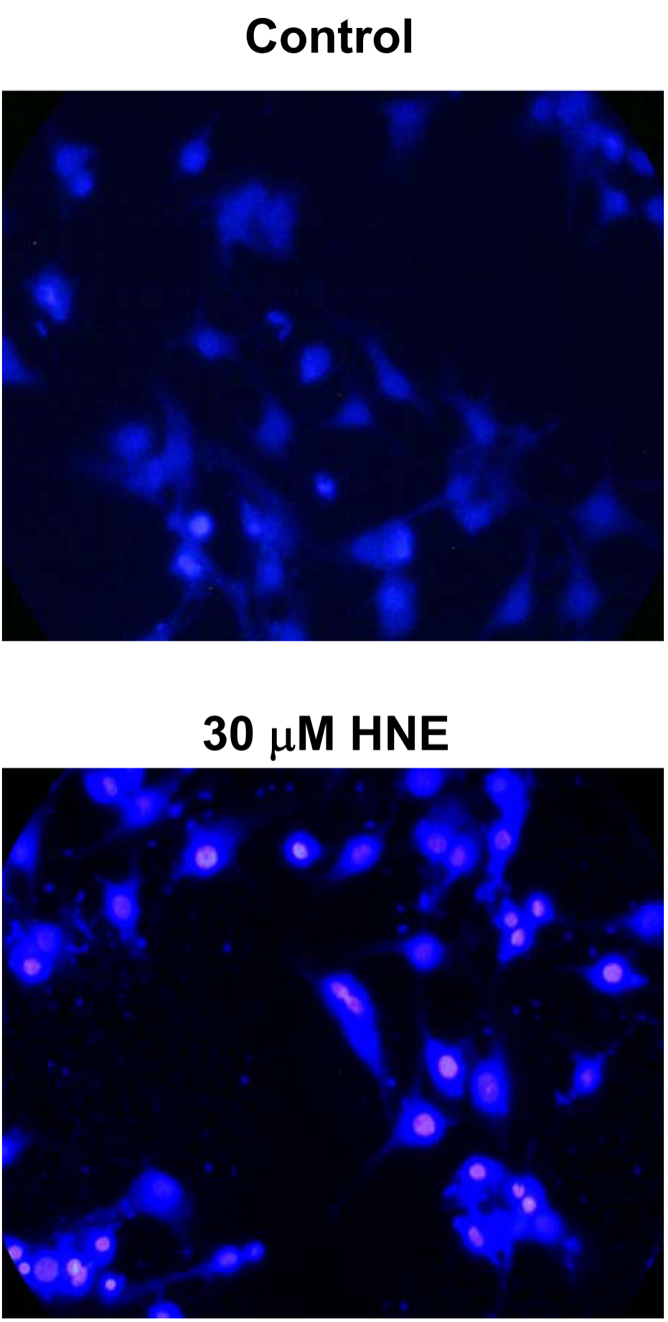
Nuclear morphology study for apoptosis. Chondrocytes were incubated for 16 hours with or without 30 μM 4-hydroxynonenal (HNE), stained with Hoechst 33258, and then analyzed by fluorescence microscopy.

To investigate the effect of HNE on chondrocyte apoptosis, we first documented HNE cytotoxicity by MTT assay. After 16 hours of incubation, up to 10 μM HNE did not alter cell viability, but 20 and 30 μM HNE was cytotoxic and significantly decreased cell viability by approximately 50% and 52%, respectively. Furthermore, pre-treatment with 200 μM NAC for 1 hour before adding 30 μM HNE (Figure 1a) completely prevented HNE-induced cell death. However, the addition of anti-Fas/CD95 (20 μg/mL) (Figure 1b) or the PARP inhibitor INH_2_BP (50 and 100 μM) (Figure 1c) 1 hour before incubation with 30 μM HNE partially prevented HNE-induced cell death. Next, Hoechst 33342 staining was undertaken to examine the morphological changes occurring to chondrocytes. Upon exposure to 30 μM HNE, numerous chondrocytes exhibited characteristics typical of apoptosis with highly condensed nuclei (Figure 2). In contrast, very few apoptotic cells were observed in untreated cells.

### HNE induced caspases activation

**Figure 3:**
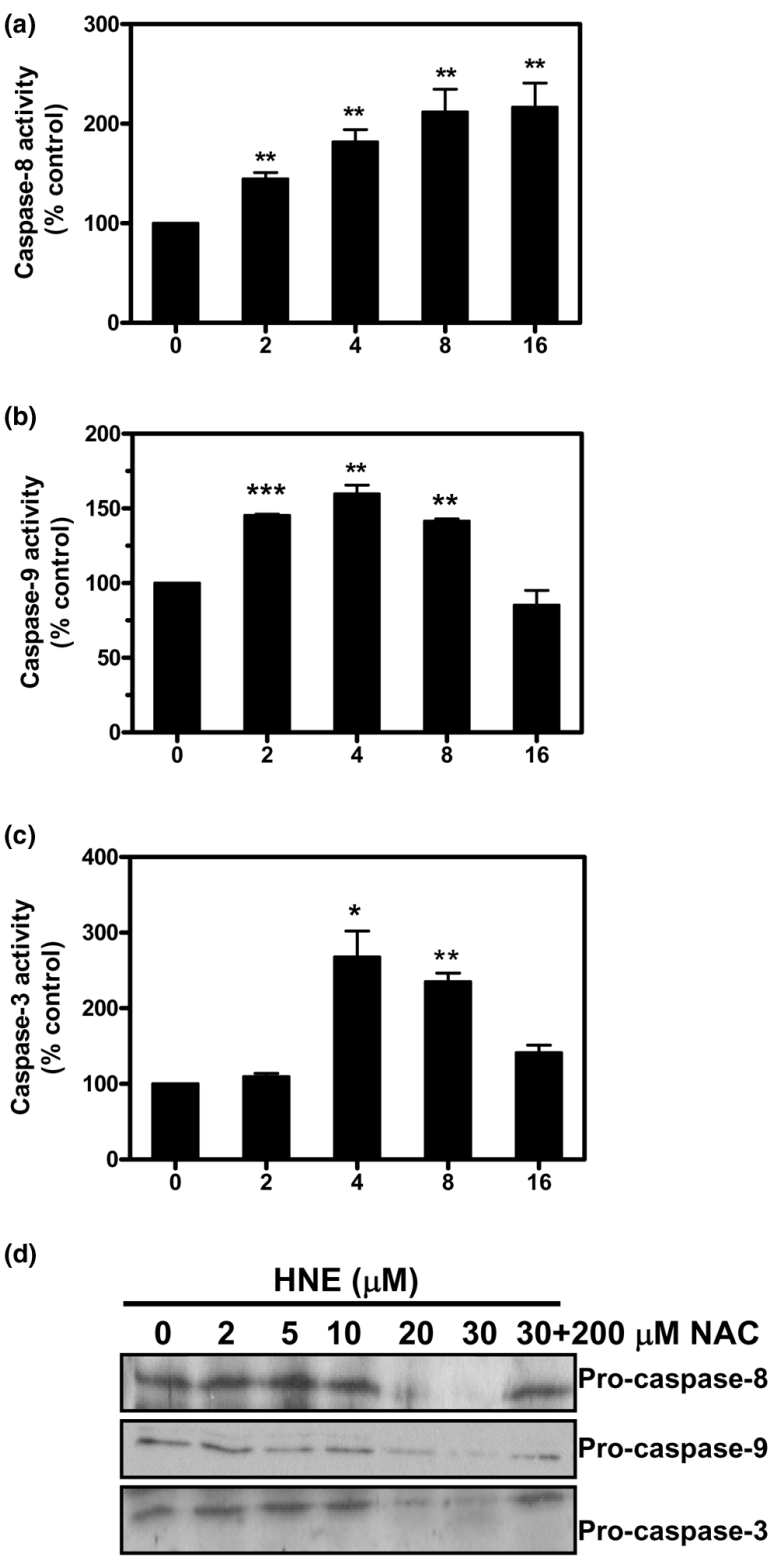
Caspase-8, -9, and -3 activation by 4-hydroxynonenal (HNE). Chondrocytes were treated with 30 μM HNE for the indicated times, and enzymatic activities of caspase-8 **(a)**, caspase-9 **(b)**, or caspase-3 **(c) **were determined with commercial kits. **(d) **Chondrocytes were pre-incubated for 1 hour with or without 200 μM *N*-acetyl-cysteine (NAC) followed by another incubation for 16 hours with increasing concentrations of HNE (0 to 30 μM). Pro-caspase-8, pro-caspase-9, and pro-caspase-3 were analyzed by Western blot. Data are mean ± standard error of the mean (n = 8) and expressed as a percentage of untreated cells. Statistics: Student unpaired *t *test; *P < 0.05, **P < 0.01, ***P < 0.001.

Caspases play important roles in the terminal execution of apoptosis induced by various stimuli. Caspase-3, -8, and -9 activities were measured with commercial kits, and their protein cleavage was detected by Western blot analysis. Compared with untreated cells, HNE incubation resulted in a time-dependent increase of caspase-8 activity (Figure 3a). HNE significantly induced caspase-9 activity after 2, 4, and 8 hours of incubation and significantly induced caspase-3 activity after 4 and 8 hours of incubation. At 16 hours, both caspase-3 and -9 activities were reduced to the control level (Figure 3b, c). At the protein level, 20 and 30 μM HNE decreased pro-caspase-8, -9, and -3 levels after 16 hours of incubation, probably via the cleavage process of the pro-caspase (Figure 3d). In contrast, the addition of 200 μM NAC prevented the HNE-induced caspase activation.

### HNE affected Bcl-2 and Bax expression and induced cytochrome c release from mitochondria

**Figure 4:**
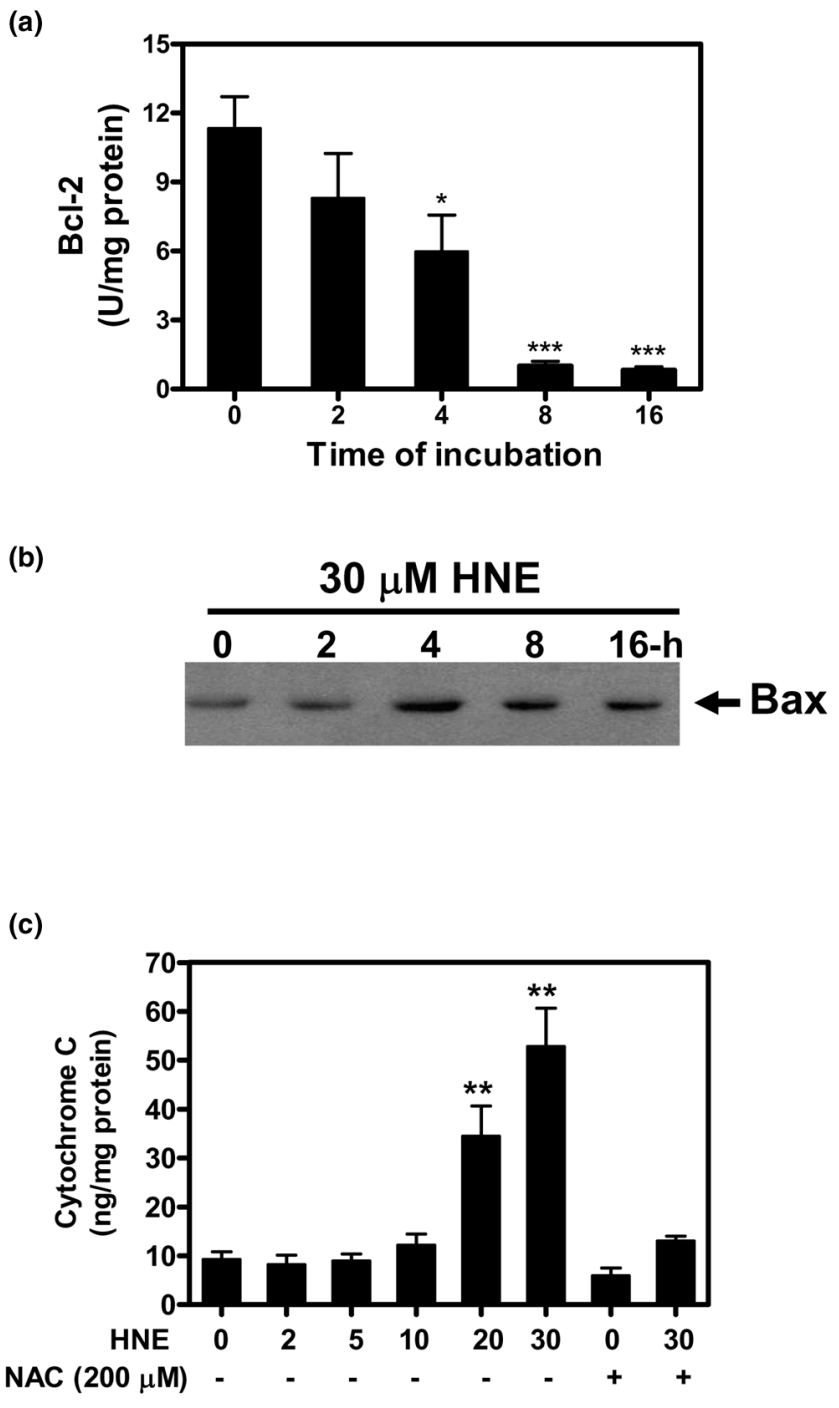
4-Hydroxynonenal (HNE) modified Bcl-2/Bax protein expression and induced cytochrome *c *release from mitochondria. Chondrocytes were treated with 30 μM HNE for the indicated times and then Bcl-2 **(a) **and Bax **(b) **protein levels were determined by commercial kit and Western blot, respectively. **(c) **Chondrocytes were pre-incubated with or without 200 μM *N*-acetyl-cysteine (NAC) for 1 hour followed by another incubation for 16 hours in the presence of increasing concentrations of HNE (0 to 30 μM). Cytochrome *c *level was assessed in cytosolic fractions with a kit. *P < 0.05, **P < 0.01, ***P < 0.001.

The Bcl-2 family is involved in apoptosis by regulating membrane permeability and induces cytochrome *c *release from mitochondria into the cytosol [22-24]. To investigate the effects of HNE on Bcl-2 and Bax expression, cells were treated with 30 μM HNE for increasing incubation times (0 to 16 hours) and then ELISA and Western blot experiments were performed. The level of the anti-apoptotic protein Bcl-2 was significantly decreased after 4 hours of incubation with 30 μM HNE (Figure 4a). In contrast, HNE at this concentration increased the apoptotic protein Bax after 4 and 8 hours of incubation and remained elevated at 16 hours of incubation (Figure 4b). We then analyzed the effect of HNE on cytochrome *c *release from mitochondria to the cytosol. As shown in Figure 4c, the cytochrome *c *level in cytosolic fractions significantly increased in chondrocytes treated with 20 or 30 μM HNE for 16 hours (Figure 4c). However, the pre-incubation of chondrocytes with 200 μM NAC prevented HNE-induced DNA fragmentation, PARP activation, and AIF translocation to the nucleus.

### HNE induced DNA fragmentation, PARP cleavage, and apoptosis-inducing factor translocation to the nucleus

**Figure 5:**
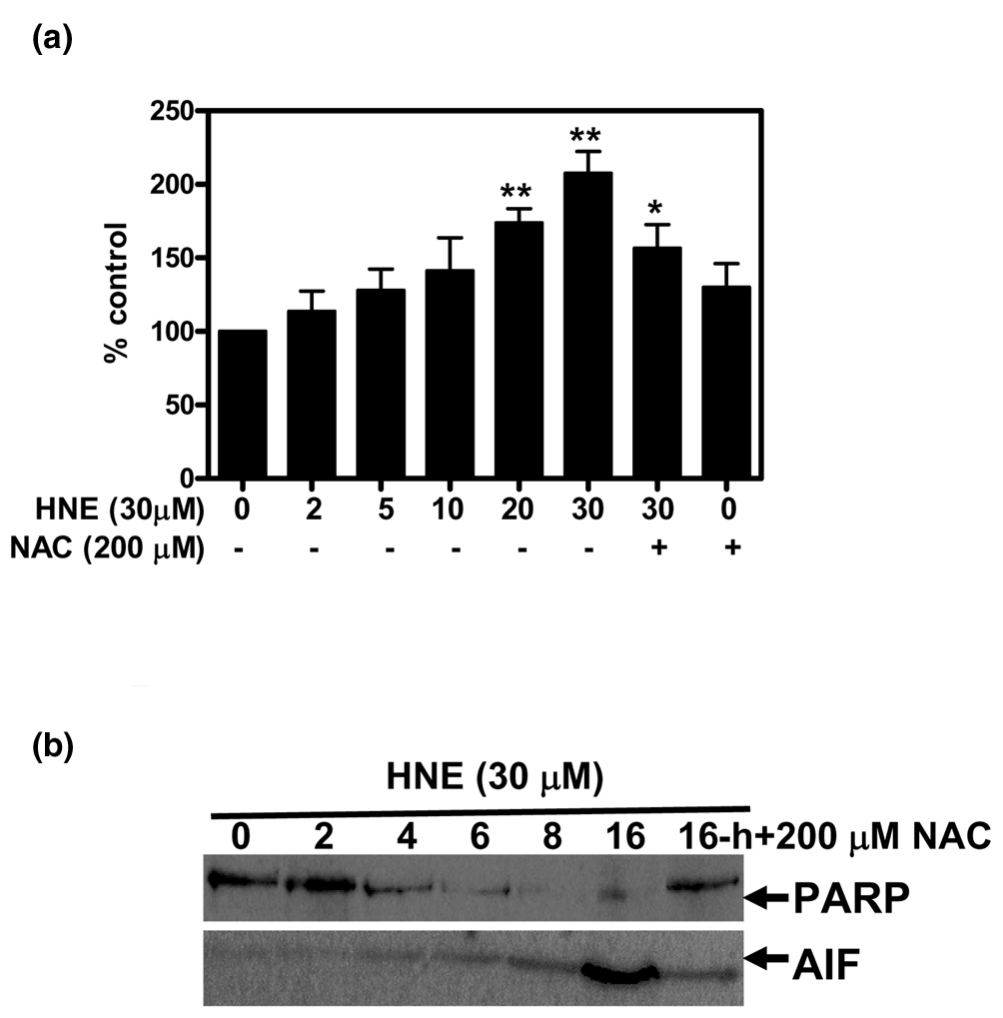
4-Hydroxynonenal (HNE) induced DNA fragmentation, poly (ADP-ribose) polymerase (PARP) cleavage, and apoptosis-inducing factor (AIF) translocation to the nucleus. Chondrocytes were pre-incubated for 1 hour with or without 200 μM *N*-acetyl-cysteine (NAC) and then incubated for another 16 hours with 30 μM HNE or with increasing concentrations of HNE (0 to 30 μM). **(a) **The cytoplasmic histone-associated DNA fragments were quantified with a kit. **(b) **Chondrocytes were pre-incubated for 1 hour with or without 200 μM NAC followed by another incubation with 30 μM HNE at different incubation times. PARP cleavage and AIF translocation in nuclear fractions were analyzed by Western blot. Data are mean ± standard error of the mean and expressed as a percentage of untreated cells. Statistics: Student unpaired *t *test; *P < 0.05, **P < 0.01.

Nuclear damage is very important in cell death. Therefore, we further studied the role of HNE in DNA fragmentation, PARP activation, and AIF translocation to the nucleus. Chondrocytes were exposed to HNE in the presence or absence of 200 μM NAC. The extent of nuclear DNA fragmentation was measured quantitatively by ELISA. As shown in Figure 5a, the level of cytoplasmic histone-associated DNA fragments was increased when DNAs were extracted from chondrocytes after exposure to 20 and 30 μM HNE for 16 hours compared with the control. In contrast, cells pre-treated with 200 μM NAC were protected against HNE-induced DNA fragmentation. PARP activation and AIF translocation to the nuclei during apoptosis are implicated on a large scale in DNA fragmentation and peripheral chromatin condensation. As shown in Figure 5b, 30 μM HNE induced PARP cleavage and AIF translocation in the nuclei after 4 hours of incubation. Moreover, pre-treatment with 200 μM NAC blocked PARP cleavage and AIF translocation.

### HNE induced Fas/CD95 and p53 expression but inhibited Akt

**Figure 6:**
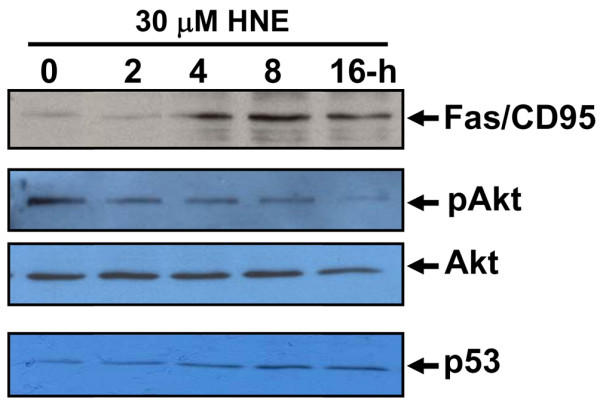
4-Hydroxynonenal (HNE) induced Fas/CD95 and p53 protein expression and reduced Akt phosphorylation. Chondrocytes were incubated with 30 μM HNE for increasing incubation times. Total cell lysates or nuclear extracts (~20 μg) were subjected to Western analysis (n = 8) using antibodies anti-Fas/CD95, anti-p53, anti-phospho Akt, and anti-total Akt.

To study the molecular mechanisms involved in the induction of cell death by HNE, we examined Akt activity as well as Fas/CD95 and p53 protein expression in chondrocytes after different incubation times. Western blot analysis indicated that 30 μM HNE elicited Fas/CD95 and p53 protein expression but, in contrast, reduced survival signalling, including the phosphorylated form of Akt (Figure 6). Total Akt was unchanged.

### HNE altered redox status and energy metabolism

**Figure 7:**
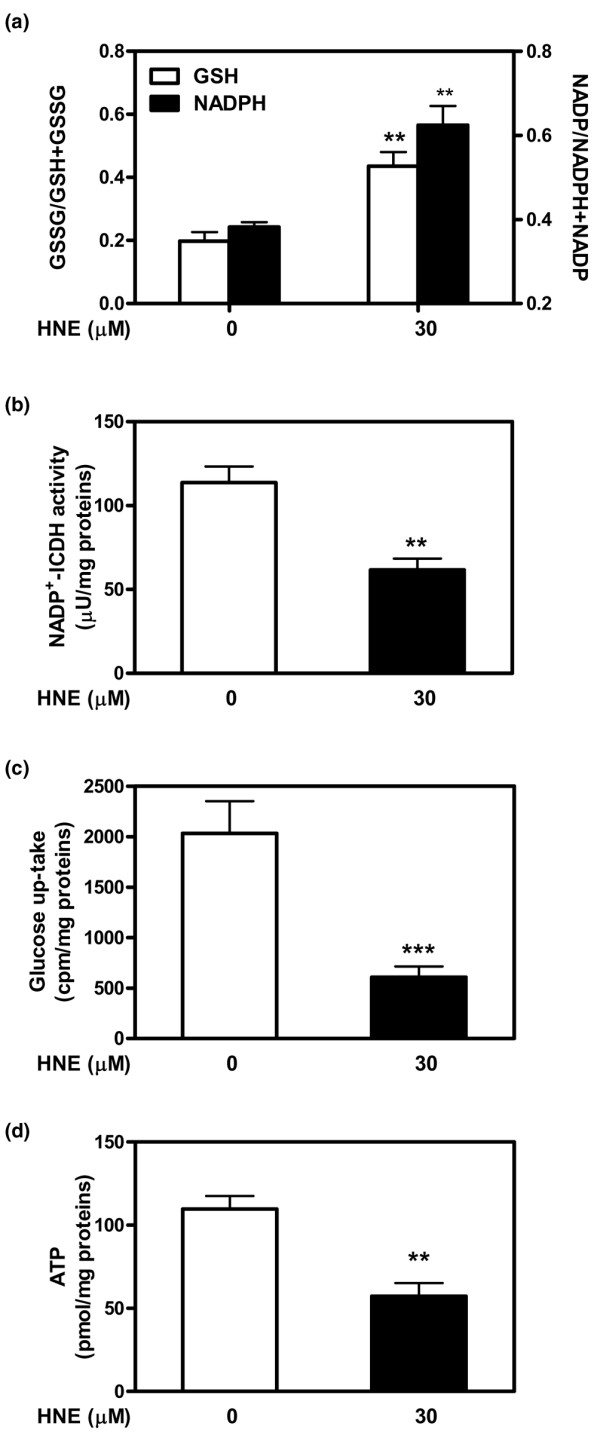
4-Hydroxynonenal (HNE) altered redox status and energy metabolism in osteoarthritis chondrocytes. Chondrocytes were treated for 16 hours with 30 μM HNE, and cellular extracts were subjected to different analysis to determine **(a) **GSSG/(GSSG+GSH) and NADP/(NADPH+NADP) ratios, **(b) **mNADP^+^-ICDH activity, **(c) **glucose uptake, and **(d) **ATP level. Data are mean ± standard error of the mean and expressed as a percentage of untreated cells. Statistics: Student unpaired *t *test; **P < 0.01, ***P < 0.001. GSH, glutathione; GSSG, oxidized glutathione; mNADP^+^-ICDH, mitochondrial NADP^+^-dependent isocitrate dehydrogenase.

In the next series of experiments, we determined whether the redox status and energy metabolism of chondrocytes could be implicated in part in HNE-induced cell death. As illustrated in Figure 7, 30 μM HNE decreased the reduced form of GSH and the reducing equivalent, NADPH, needed for its regeneration (Figure 7a), after 16 hours of incubation. We additionally established that HNE, at this concentration, evoked a significant diminution of energy depletion through inhibition of mNADP^+^-ICDH activity (Figure 7b), glucose uptake (Figure 7c), and intracellular ATP synthesis (Figure 7d).

### HNE-induced cell death is controlled by GSTA4-4 expression

**Figure 8:**
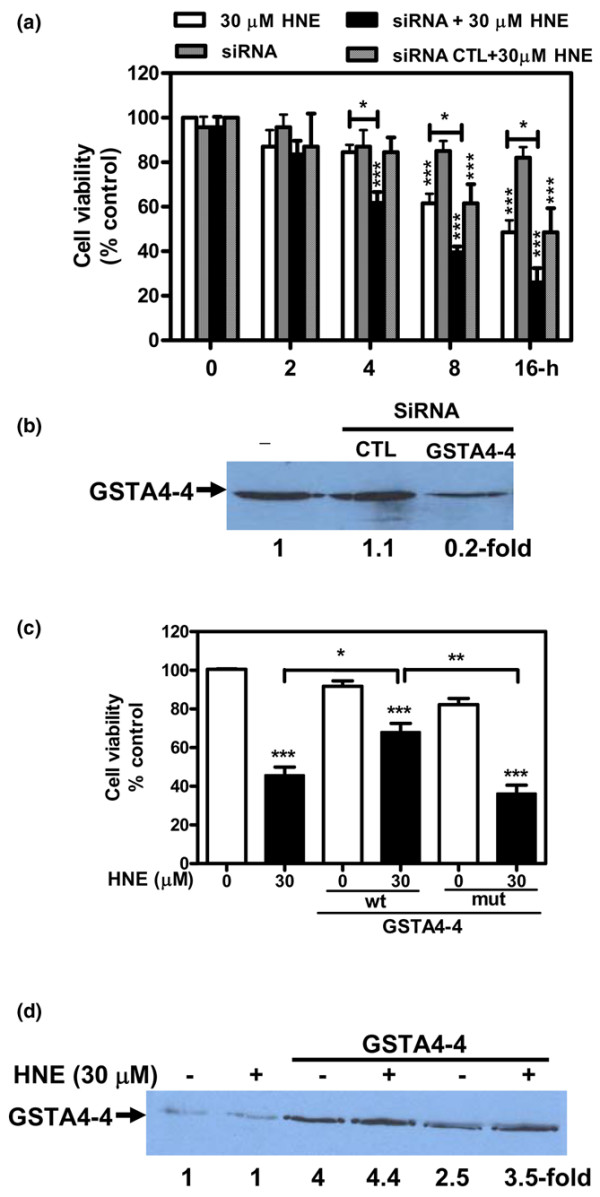
Glutathione-S-transferase A4-4 (GSTA4-4) controlled the 4-hydroxynonenal (HNE) cytotoxicity. Chondrocytes were transfected with **(a) **small interfering RNA (siRNA) GSTA4-4 or with **(c) **wild-type or mutant GSTA4-4 expression plasmids, and then cell viability was determined by MTT assay. **(b) and (d) **GSTA4-4 protein expression was evaluated respectively in cellular extracts of transfected chondrocytes with siRNA or expression plasmids of GSTA4-4 by Western blotting. Data are mean ± standard error of the mean and expressed as a percentage of untreated cells. Statistics: Student unpaired *t *test; *P < 0.05, **P < 0.01, ***P < 0.001. CTL, control; MTT, 3-(4,5-dimethylthiazol-2-yl)-2,5-diphenyltetrazolium bromide; mut, mutant; wt, wild-type.

GSTA4-4 is a known aldehyde-detoxifying enzyme as has been shown by previous studies [16,17]. To assess the functional consequences of GSTA4-4 inhibition versus overexpression in chondrocytes, the cytotoxicity of 30 μM HNE was evaluated by MTT cytotoxicity assay. First, the ablation of GSTA4-4 with GSTA4-4 siRNA in isolated chondrocytes augmented the HNE-induced cell mortality as measured by MTT assay at 4, 8, and 16 hours of incubation (Figure 8a). These results indicate that GSTA4-4 offers a significant protection against HNE-induced DNA damage in chondrocyte cells and that siRNA ablation of this enzyme augments the HNE-induced cell death. The increase in cell mortality in transfected chondrocytes with GSTA4-4 siRNA would be attributed to the inhibition of GSTA4-4 expression by more than 80% as compared with control siRNA (Figure 8b). Second, we tested whether the increased HNE-metabolizing capacity conferred on these cells by transfection of GSTA4-4 expression vectors could reverse the cytotoxic effects of HNE. Our data showed that GSTA4-4 overexpression provided cell resistance to direct HNE cytotoxicity (Figure 8c). Western blot analysis of cell extracts with the polyclonal antibody against GSTA4-4 revealed a strong band in cellular extracts of chondrocytes transfected with GSTA4-4 and a weak signal in untransfected cells (Figure 8d).

## Discussion

There is growing evidence that HNE, generated during the LPO process, is an efficient cell signalling molecule and considered as a key mediator of oxidative stress-induced pathophysiological effects. In fact, by modulating the expression of different genes, HNE exhibits a wide array of biological activities, including signal transduction, gene expression, and modulation of cell proliferation [13]. In particular, the relevance of HNE to joint biology and pathology is now becoming clearer. In our previous study, we reported, for the first time, that HNE was significantly increased in synovial fluids of OA patients and in OA chondrocytes treated with ROS donors [18]. We demonstrated that 10 μM HNE affects Col II and MMP-13 at the transcriptional and post-translational levels, indicating that HNE could play a role in cartilage degradation in OA. In a second study, we identified two distinct mechanisms of HNE action in OA chondrocytes, which included upregulation of COX-2 via ATF/CRE activation and iNOS inhibition via NF-κB inactivation [19]. In the present study, we evaluated the potential role of HNE at high concentrations in the apoptotic process in OA chondrocytes. With the ultimate goal of clarifying this role, we documented the ability of HNE to modulate markers of apoptosis, redox status, and energy metabolism of chondrocytes. Then, we investigated the potential role of the HNE-metabolizing enzyme, GSTA4-4, in the control of chondrocyte death. To date, no data have demonstrated the effect of HNE on apoptotic signalling in OA.

In the present study, we reported morphological and biochemical evidence implicating HNE in the induction of chondrocyte apoptosis in a dose- and time-dependent manner, and this effect could be inhibited by NAC addition. First, we observed that HNE up to 10 μM did not alter cell viability but equal or greater than to 20 μM, HNE was cytotoxic and significantly decreased cell viability (up to 50%) and induced chromatin condensation, compared with untreated cells. Pre-treatment of cells with anti-Fas/CD95 or PARP inhibitor partially reduced cell mortality, suggesting a role for Fas/CD95 and PARP in HNE-inducing cell death. Second, in investigating the classical markers of apoptosis, we obtained data showing that HNE induced caspase-8, -9, and -3 activities and cleavage, AIF and cytochrome *c *release from mitochondria, PARP activation, and DNA fragmentation. These effects were prevented by NAC. A major question arising with regard to apoptosis in general is whether the apoptotic response of chondrocytes to HNE requires upregulation of pro-apoptotic protein synthesis or whether it relies on pre-existing apoptotic machinery. Our data show that the apoptotic response of chondrocytes to HNE is associated with decreased Bcl-2 expression and increased Bax expression. This suggests that chondrocytes need to modulate the synthesis of at least several anti- and pro-apototic factors to be able to undergo apoptosis when stimulated by HNE. It is noteworthy that HNE is capable of inducing apoptosis in several cell types, including hepatic cells, murine alveolar macrophages, RAW 264.7 cells, neurons as well as colonic cancer cells [15,25,26]. It is well documented that these cells upregulate pro-apoptotic factors (for example, caspases, PARP, Bax, and Bcl-2) to undergo apoptosis in response to HNE. The DNA fragmentation evoked by HNE required PARP activation and AIF translocation in the nucleus [27,28]. Moreover, our data disclosed that HNE-induced apoptosis in chondrocytes needed the induction of p53 and Fas/CD95 protein expression in a time-dependent manner. These data are in concordance with those in the literature suggesting that HNE can induce apoptosis in various cells through the death receptor Fas/CD95-mediated extrinsic pathway as well as through the p53-dependent intrinsic pathway [29,30]. The induction of Fas/CD95 by HNE was subsequently followed by the activation of JNKs, caspase 3, and the onset of apoptosis in human lens epithelial cells [29]. However, the induction of p53 by HNE remains unclear. In RAW 264.7 cells, Haynes and colleagues [25] reported that HNE-induced apoptosis was not associated with p53 accumulation. This suggests that the apoptosis process elicited by HNE could vary in different cell types due to changes in the availability or sensitivity of specific regulatory pathways that are activated by the aldehyde.

Investigation of the molecular mechanisms of HNE-induced apoptosis in OA chondrocytes showed that HNE reduced serine/theonine kinase Akt activity. It has also been established that HNE-induced apoptosis leads to the inhibition of Akt phosphorylation. Akt activation results in the phosphorylation of numerous other proteins involved in the regulation of glucose metabolism, cell proliferation, apoptosis, cell migration, and gene expression [31]. HNE is definitely able to interfere with Akt activity, but so far the available findings appear, at least in part, to be contradictory. In fact, Liu and colleagues [32] reported that HNE (20 μM) induced apoptosis in human T-cell leukemia Jurkat cells through impairment of the Akt-mediated cell survival pathway.

The next set of experiments was focused on analyzing redox status and cell metabolism in apoptotic chondrocytes. Significant inhibition of cellular GSH pools, mNADP^+^-ICDH activity, glucose uptake, and ATP level was observed. Liu and colleagues [27] actually showed that exogenously added HNE quickly reduced cellular GSH levels in human T-lymphoma Jurkat cells. Parallelling the change in GSH levels, GSSG levels decreased, suggesting that HNE is directly reacting with GSH for consumption rather than acting as a source of pro-oxidants to simply promote GSH/GSSG exchange. It is, however, also possible that HNE decreased the GSH pool through inhibition of GSH synthesis. In any case, pre-treatment of cells with antioxidants, such as cysteine, NAC, and dithiothreitol, inhibited the action of HNE to reduce the GSH/GSSG pool, supporting the view that SH group-reactive HNE activity is primarily important for the observed event. On the other hand, the inhibition of mNADP^+^-ICDH, an important regulator of the citric acid cycle, by HNE supports the role of this aldehyde in the alteration of energy metabolism in different cell types as reported by the literature data. Our previous study revealed that mNADP^+^-ICDH was considered as a potential target for HNE binding [33]. This key enzyme in cellular defence against oxidative damage supplies NADPH in the mitochondria needed for the regeneration of mitochondrial GSH [34]. In the present study, we further demonstrated HNE cytotoxicity in chondrocytes presumably via ATP depletion caused by the inhibition of mitochondrial respiratory enzymes and glucose uptake. Glucose serves as the major energy substrate for articular chondrocytes and as the main precursor for the synthesis of ECM glycosaminoglycans in cartilage [35,36]. The inhibition of proteoglycan synthesis in OA chondrocytes by HNE (data not shown) could be attributed to glucose uptake blockage observed in the present work. ATP is the main energy source for cellular proliferation, differentiation, and apoptosis [37,38]. Kanwar and colleagues [39] demonstrated that cellular ATP depletion markedly decreased the synthesis of sulfated proteoglycans by a mechanism that was normalized by increasing cellular ATP levels.

To determine whether HNE-metabolizing enzymes played a role in chondrocyte apoptosis, we conducted additional experiments to show that the changes in GSTA4-4 expression could have an impact in HNE-induced cell death. In chondrocytes, GSTA4-4 ablation with GSTA4-4 siRNA augmented the cytotoxic effect of HNE as determined by MTT assay. In contrast, the overexpression of this enzyme in chondrocytes offered significant protection against HNE-induced cell cytotoxicity. These data concur with a previous report indicating that accelerated HNE metabolism in HL-60 and K562 human erythroleukemia cells overexpressing GSTA4-4 blocks/delays apoptosis triggered by HNE [16,17]. Increased HNE-metabolizing capacity in GSTA4-4-transfected cells appeared to result in an increased rate of proliferation as well as decreased cell death by preventing PARP and caspase activation. Transfection of GSTA4-4 also protected against cytotoxic H_2_O_2 _cytotoxicity to a similar degree, an effect that could be ascribed either to the direct enzymatic detoxification of H_2_O_2 _through the GSH-peroxidase activity of GSTA4-4 or to increased metabolism via GSH conjugation of HNE formed as a consequence of H_2_O_2 _exposure.

## Conclusion

In this study, we identified, for the first time, a novel mechanism linking oxidative stress to apoptosis signalling in OA chondrocytes through the action of HNE, an LPO end product. Our data suggest that the increased level of HNE in articular tissue may contribute to OA development via its ability to alter cellular viability and the metabolic activity of chondrocytes. In the light of previous data on decreased GSTA4-4 activity in patients with OA, particular interest should be addressed to the pathophysiological role of this enzyme in OA development.

## Abbreviations

AIF: apoptosis-inducing factor; ATF/CRE: activating transcription factor/cAMP response element; Col II: type II collagen; COX-2: cyclooxygenase-2; DMEM: Dulbecco's modified Eagle's medium; ECM: extracellular matrix; EDTA: ethylenediaminetetraacetic acid; ELISA: enzyme-linked immunosorbent assay; FBS: fetal bovine serum; GSH: glutathione; GSSG: oxidized glutathione; GSTA4-4: glutathione-S-transferase A4-4; HNE: 4-hydroxynonenal; iNOS: inducible nitric oxide synthase; LPO: lipid peroxidation; MMP-13: matrix metalloproteinase-13; mNADP^+^-ICDH: mitochondrial NADP^+^-dependent isocitrate dehydrogenase; MTT: 3-(4,5-dimethylthiazol-2-yl)-2,5-diphenyltetrazolium bromide; NAC: *N*-acetyl-cysteine; NF-κB: nuclear factor-kappa B; NO: nitric oxide; OA: osteoarthritis; PARP: poly (ADP-ribose) polymerase; PBS: phosphate-buffered saline; ROS: reactive oxygen species; siRNA: small interfering RNA.

## Competing interests

The authors declare that they have no competing interests.

## Authors' contributions

FV performed the experimental study, contributed to the preparation of the manuscript, and undertook the statistical analysis. HF, PL, and JCF evaluated and interpreted the data and assisted with the preparation of the manuscript. QS assisted in the experiments and in the isolation of chondrocytes from human cartilage. PR provided cartilage specimens and participated to the study design. MB designed the study, supervised the project, evaluated and interpreted the data, and prepared the manuscript. All authors read and approved the final manuscript.

## References

[B1] Poole AR (1999). An introduction to the pathophysiology of osteoarthritis. Front Biosci.

[B2] Sandell LJ, Aigner T (2001). Articular cartilage and changes in arthritis. An introduction: cell biology of osteoarthritis. Arthritis Res.

[B3] Blanco FJ, Guitian R, Vazquez-Martul E, de Toro FJ, Galdo F (1998). Osteoarthritis chondrocytes die by apoptosis. A possible pathway for osteoarthritis pathology. Arthritis Rheum.

[B4] Kim HA, Lee YJ, Seong SC, Choe KW, Song YW (2000). Apoptotic chondrocyte death in human osteoarthritis. J Rheumatol.

[B5] Mistry D, Oue Y, Chambers MG, Kayser MV, Mason RM (2004). Chondrocyte death during murine osteoarthritis. Osteoarthritis Cartilage.

[B6] Hashimoto S, Ochs RL, Komiya S, Lotz M (1998). Linkage of chondrocyte apoptosis and cartilage degradation in human osteoarthritis. Arthritis Rheum.

[B7] Murrell GA, Jang D, Williams RJ (1995). Nitric oxide activates metalloprotease enzymes in articular cartilage. Biochem Biophys Res Commun.

[B8] Taskiran D, Stefanovic-Racic M, Georgescu H, Evans C (1994). Nitric oxide mediates suppression of cartilage proteoglycan synthesis by interleukin-1. Biochem Biophys Res Commun.

[B9] Blanco FJ, Ochs RL, Schwarz H, Lotz M (1995). Chondrocyte apoptosis induced by nitric oxide. Am J Pathol.

[B10] Del CM, Loeser RF (2002). Nitric oxide-mediated chondrocyte cell death requires the generation of additional reactive oxygen species. Arthritis Rheum.

[B11] Hashimoto S, Takahashi K, Amiel D, Coutts RD, Lotz M (1998). Chondrocyte apoptosis and nitric oxide production during experimentally induced osteoarthritis. Arthritis Rheum.

[B12] Esterbauer H, Schaur RJ, Zollner H (1991). Chemistry and biochemistry of 4-hydroxynonenal, malonaldehyde and related aldehydes. Free Radic Biol Med.

[B13] Poli G, Schaur RJ, Siems WG, Leonarduzzi G (2008). 4-Hydroxynonenal: a membrane lipid oxidation product of medicinal interest. Med Res Rev.

[B14] Barrera G, Pizzimenti S, Laurora S, Briatore F, Toaldo C, Dianzani MU (2005). 4-hydroxynonenal and cell cycle. Biofactors.

[B15] Cerbone A, Toaldo C, Laurora S, Briatore F, Pizzimenti S, Dianzani MU, Ferretti C, Barrera G (2007). 4-Hydroxynonenal and PPARgamma ligands affect proliferation, differentiation, and apoptosis in colon cancer cells. Free Radic Biol Med.

[B16] Cheng JZ, Singhal SS, Saini M, Singhal J, Piper JT, Van Kuijk FJ, Zimniak P, Awasthi YC, Awasthi S (1999). Effects of mGST A4 transfection on 4-hydroxynonenal-mediated apoptosis and differentiation of K562 human erythroleukemia cells. Arch Biochem Biophys.

[B17] Cheng JZ, Singhal SS, Sharma A, Saini M, Yang Y, Awasthi S, Zimniak P, Awasthi YC (2001). Transfection of mGSTA4 in HL-60 cells protects against 4-hydroxynonenal-induced apoptosis by inhibiting JNK-mediated signaling. Arch Biochem Biophys.

[B18] Morquette B, Shi Q, Lavigne P, Ranger P, Fernandes JC, Benderdour M (2006). Production of lipid peroxidation products in osteoarthritic tissues: new evidence linking 4-hydroxynonenal to cartilage degradation. Arthritis Rheum.

[B19] Vaillancourt F, Morquette B, Shi Q, Fahmi H, Lavigne P, Di Battista JA, Fernandes JC, Benderdour M (2007). Differential regulation of cyclooxygenase-2 and inducible nitric oxide synthase by 4-hydroxynonenal in human osteoarthritic chondrocytes through ATF-2/CREB-1 transactivation and concomitant inhibition of NF-kappaB signaling cascade. J Cell Biochem.

[B20] Altman R, Asch E, Bloch D, Bole G, Borenstein D, Brandt K, Christy W, Cooke TD, Greenwald R, Hochberg M (1986). Development of criteria for the classification and reporting of osteoarthritis. Classification of osteoarthritis of the knee. Diagnostic and Therapeutic Criteria Committee of the American Rheumatism Association. Arthritis Rheum.

[B21] Mosmann T (1983). Rapid colorimetric assay for cellular growth and survival: application to proliferation and cytotoxicity assays. J Immunol Methods.

[B22] Cory S, Adams JM (2002). The Bcl2 family: regulators of the cellular life-or-death switch. Nat Rev Cancer.

[B23] Rosse T, Olivier R, Monney L, Rager M, Conus S, Fellay I, Jansen B, Borner C (1998). Bcl-2 prolongs cell survival after Bax-induced release of cytochrome *c*. Nature.

[B24] Kluck RM, Bossy-Wetzel E, Green DR, Newmeyer DD (1997). The release of cytochrome *c *from mitochondria: a primary site for Bcl-2 regulation of apoptosis. Science.

[B25] Haynes RL, Brune B, Townsend AJ (2001). Apoptosis in RAW 264.7 cells exposed to 4-hydroxy-2-nonenal: dependence on cytochrome *C *release but not p53 accumulation. Free Radic Biol Med.

[B26] Peng ZF, Koh CH, Li QT, Manikandan J, Melendez AJ, Tang SY, Halliwell B, Cheung NS (2007). Deciphering the mechanism of HNE-induced apoptosis in cultured murine cortical neurons: transcriptional responses and cellular pathways. Neuropharmacology.

[B27] Liu W, Kato M, Akhand AA, Hayakawa A, Suzuki H, Miyata T, Kurokawa K, Hotta Y, Ishikawa N, Nakashima I (2000). 4-hydroxynonenal induces a cellular redox status-related activation of the caspase cascade for apoptotic cell death. J Cell Sci.

[B28] Ramachandran V, Perez A, Chen J, Senthil D, Schenker S, Henderson GI (2001). *In utero *ethanol exposure causes mitochondrial dysfunction, which can result in apoptotic cell death in fetal brain: a potential role for 4-hydroxynonenal. Alcohol Clin Exp Res.

[B29] Li J, Sharma R, Patrick B, Sharma A, Jeyabal PV, Reddy PM, Saini MK, Dwivedi S, Dhanani S, Ansari NH, Zimniak P, Awasthi S, Awasthi YC (2006). Regulation of CD95 (Fas) expression and Fas-mediated apoptotic signaling in HLE B-3 cells by 4-hydroxynonenal. Biochemistry.

[B30] Awasthi YC, Sharma R, Sharma A, Yadav S, Singhal SS, Chaudary P, Awasthi S (2008). Self-regulatory role of 4-hydroxynonenal in signaling for stress-induced programmed cell death. Free Radic Biol Med.

[B31] Brazil DP, Hemmings BA (2001). Ten years of protein kinase B signalling: a hard Akt to follow. Trends Biochem Sci.

[B32] Liu W, Akhand AA, Takeda K, Kawamoto Y, Itoigawa M, Kato M, Suzuki H, Ishikawa N, Nakashima I (2003). Protein phosphatase 2A-linked and -unlinked caspase-dependent pathways for downregulation of Akt kinase triggered by 4-hydroxynonenal. Cell Death Differ.

[B33] Benderdour M, Charron G, Deblois D, Comte B, Des Rosiers C (2003). Cardiac mitochondrial NADP^+^-isocitrate dehydrogenase is inactivated through 4-hydroxynonenal adduct formation: an event that precedes hypertrophy development. J Biol Chem.

[B34] Jo SH, Son MK, Koh HJ, Lee SM, Song IH, Kim YO, Lee YS, Jeong KS, Kim WB, Park JW, Song BJ, Huh TL (2001). Control of mitochondrial redox balance and cellular defense against oxidative damage by mitochondrial NADP^+^-dependent isocitrate dehydrogenase. J Biol Chem.

[B35] Wang J, Zhou J, Bondy CA (1999). Igf1 promotes longitudinal bone growth by insulin-like actions augmenting chondrocyte hypertrophy. FASEB J.

[B36] Mobasheri A, Vannucci SJ, Bondy CA, Carter SD, Innes JF, Arteaga MF, Trujillo E, Ferraz I, Shakibaei M, Martin-Vasallo P (2002). Glucose transport and metabolism in chondrocytes: a key to understanding chondrogenesis, skeletal development and cartilage degradation in osteoarthritis. Histol Histopathol.

[B37] Agresti C, Meomartini ME, Amadio S, Ambrosini E, Volonte C, Aloisi F, Visentin S (2005). ATP regulates oligodendrocyte progenitor migration, proliferation, and differentiation: involvement of metabotropic P2 receptors. Brain Res Brain Res Rev.

[B38] Bernardi P, Scorrano L, Colonna R, Petronilli V, Di Lisa F (1999). Mitochondria and cell death. Mechanistic aspects and methodological issues. Eur J Biochem.

[B39] Kanwar Y, Yoshinaga Y, Liu Z, Wallner E, Carone F (1992). Biosynthetic regulation of proteoglycans by aldohexoses and ATP. Proc Natl Acad Sci USA.

